# Supplementary material for: The Small RNA Universe of Capitella teleta
Source: Front Mol Biosci. 2022 Feb 25;9:802814. doi: 10.3389/fmolb.2022.802814 (PMC8915122; doi:10.3389/fmolb.2022.802814)
Supplement: Supplementary file 1 [file DataSheet1.ZIP › Supplement/known/CAPTEscaffold_43_4499.pdf]

Provisional ID : CAPTEscaffold_43_4499
Score total : 585333
Score for star read(s) : 3.9
Score for read counts : 585322.7
Score for mfe : 1.8
Score for randfold : 1.6
Score for cons. seed : 3
Total read count : 1148097
Mature read count : 1145116
Loop read count : 1
Star read count : 2980

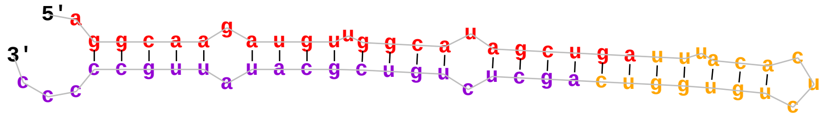

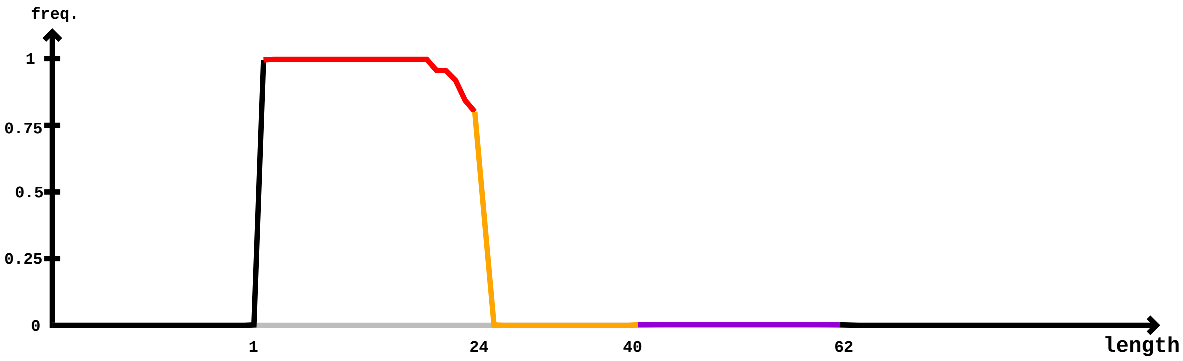

Mature   Star

```
5'- gcgaucucagggcacagaccaggcaagauguuggcauagcugauuuacacucugugguc agcucugucgcauauugcccccucugggcccagacuaacaucuuaugugaaaca -3'   obs
```

```
5'- gcgaucucagggcacagaccaggcaagauguuggcauagcugauuuacacucuguggucagcucugucgcauauugcccccucugggcccagacuaacaucuuaugugaaaca -3'   obs
    gcgaucucagggcacagaccaggcaagauguuggcauagcugauuuacacucuguggucagcucugucgcauauugcccccucugggcccagacuaacaucuuaugugaaaca       exp
    gcgaucucagggcacagaccaggcaagauguuggcauagcugauuuacacucuguggucagcucugucgcauauugcccccucugggcccagacuaacaucuuaugugaaaca       known
    ......((.((((.((((...(((((.((((.((((.((((((((.(((...))))))))))).)))))))).)))))...)))).)))).))...((((...))))......    reads   mm   sample

    .................aUcaggcaagauguuggcauagcuga......................................................................    2       1    seq
    ..................Ucaggcaagauguuggcauagcuga......................................................................    4       1    seq
    ...................caggcaagauguuggcaua...........................................................................    56      0    seq
    ...................caggAaagauguuggcaua...........................................................................    1       1    seq
    ...................caggcGagauguuggcauagc.........................................................................    1       1    seq
    ...................caggcaagauguuggcauagc.........................................................................    85      0    seq
    ...................Uaggcaagauguuggcauagc.........................................................................    3       1    seq
    ...................caggcaagauguAggcauagcu........................................................................    1       1    seq
    ...................caggcaagauguuggcauagcu........................................................................    299     0    seq
    ...................caggcaGgauguuggcauagcu........................................................................    1       1    seq
    ...................caggcaagauguuCgcauagcu........................................................................    1       1    seq
    ...................caggcaagauguuggcauagcug.......................................................................    610     0    seq
    ...................caggcaagauguuggcauagcuA.......................................................................    5       1    seq
    ...................Uaggcaagauguuggcauagcug.......................................................................    1       1    seq
    ...................caggcaagauguuggcauagcGg.......................................................................    1       1    seq
    ...................caggcaagauguuggcauagcAg.......................................................................    1       1    seq
    ...................caggcaagauguuggcauagcuU.......................................................................    45      1    seq
    ...................caggcaaUauguuggcauagcug.......................................................................    1       1    seq
    ...................caggcaagauguuAgcauagcug.......................................................................    1       1    seq
    ...................Aaggcaagauguuggcauagcuga......................................................................    6       1    seq
    ...................caggcaagauguuggcauagcuCa......................................................................    1       1    seq
    ...................caggcaagauguuggcauagcuga......................................................................    228     0    seq
    ...................Uaggcaagauguuggcauagcuga......................................................................    84      1    seq
    ...................caggcaagauguuggcauagcugU......................................................................    222     1    seq
    ...................caggcaagauguuggcauagcugG......................................................................    2       1    seq
    ...................caggcaagauguuggcauagcuAa......................................................................    1       1    seq
    ...................Uaggcaagauguuggcauagcugau.....................................................................    2       1    seq
    ...................caggcaagauguuggcauagcugUu.....................................................................    46      1    seq
    ....................aggcaagauguugAcaua...........................................................................    5       1    seq
    ....................aggcaagauguuggcaua...........................................................................    46544   0    seq
    ....................aggcaaAauguuggcaua...........................................................................    2       1    seq
```

Mature **Star**

```
gcgaucucagggcacagaccaggcaagauguuggcauagcugauuuacacucugugggucagcucugucgcauauugcccccucugggcccagacuaacaucuuaugugaaaca
....................aggcaagauguuggcaAa...........................................................................  8     1  seq
....................aggcaagauguuUgcaua...........................................................................  3     1  seq
....................aggcaagauguuggcauG...........................................................................  12    1  seq
....................agUcaagauguuggcaua...........................................................................  9     1  seq
....................aggcaagauguuggcauC...........................................................................  1     1  seq
....................aAgcaagauguuggcaua...........................................................................  19    1  seq
....................aggcaagauguuggcaCa...........................................................................  5     1  seq
....................aggcaagauAuuggcaua...........................................................................  6     1  seq
....................aggcaagauguuggUaua...........................................................................  10    1  seq
....................aggcaagauguuCgcaua...........................................................................  5     1  seq
....................aggcaagauguuggcGua...........................................................................  11    1  seq
....................aCgcaagauguuggcaua...........................................................................  12    1  seq
....................aggcaagaAguuggcaua...........................................................................  7     1  seq
....................aggcaagauguuggAaua...........................................................................  12    1  seq
....................aggcaagauguuggcaGa...........................................................................  5     1  seq
....................aggcaagauUuuggcaua...........................................................................  3     1  seq
....................aggUaagauguuggcaua...........................................................................  6     1  seq
....................aggcaagauguuggcUua...........................................................................  5     1  seq
....................aggcaGgauguuggcaua...........................................................................  5     1  seq
....................aggcaagauguuAgcaua...........................................................................  30    1  seq
....................aggcaagauguuggcauU...........................................................................  79    1  seq
....................aNgcaagauguuggcaua...........................................................................  2     1  seq
....................aggcaagGuguuggcaua...........................................................................  5     1  seq
....................aggcaagauguuggGaua...........................................................................  10    1  seq
....................aggcaagaugAuggcaua...........................................................................  19    1  seq
....................aggcaagauCuuggcaua...........................................................................  5     1  seq
....................aggcaagauguCggcaua...........................................................................  6     1  seq
....................aggcaagauguGggcaua...........................................................................  3     1  seq
....................aggcaagauguugUcaua...........................................................................  6     1  seq
....................aggcaagauguuggcCua...........................................................................  1     1  seq
....................aUgcaagauguuggcaua...........................................................................  4     1  seq
....................aggcGagauguuggcaua...........................................................................  10    1  seq
....................agAcaagauguuggcaua...........................................................................  14    1  seq
....................agCcaagauguuggcaua...........................................................................  15    1  seq
....................aggcaagUuguuggcaua...........................................................................  4     1  seq
....................Gggcaagauguuggcaua...........................................................................  143   1  seq
....................aggcaaCauguuggcaua...........................................................................  2     1  seq
....................aggcaagaGguuggcaua...........................................................................  2     1  seq
....................aggcaagauguAggcaua...........................................................................  28    1  seq
....................aggcaagauguugCcaua...........................................................................  1     1  seq
....................aggGaagauguuggcaua...........................................................................  4     1  seq
....................aggAaagauguuggcaua...........................................................................  11    1  seq
....................aggcaagaCguuggcaua...........................................................................  3     1  seq
....................aggcaaUauguuggcaua...........................................................................  3     1  seq
....................aggcaagaugCuggcaua...........................................................................  3     1  seq
....................Uggcaagauguuggcaua...........................................................................  2     1  seq
....................aggcaUgauguuggcaua...........................................................................  5     1  seq
....................aggcUagauguuggcaua...........................................................................  3     1  seq
....................Nggcaagauguuggcaua...........................................................................  15    1  seq
....................aggcaagaugAuggcauag..........................................................................  1     1  seq
....................aggcaagauguuggcGuag..........................................................................  1     1  seq
....................aggcaagauguuggcauaA..........................................................................  25    1  seq
....................Nggcaagauguuggcauag..........................................................................  1     1  seq
....................Gggcaagauguuggcauag..........................................................................  6     1  seq
....................aggcaagauguuggcaAag..........................................................................  1     1  seq
....................aggcaagaAguuggcauag..........................................................................  1     1  seq
....................aggAaagauguuggcauag..........................................................................  1     1  seq
....................aggcaagauguuggGauag..........................................................................  2     1  seq
....................aggcaagaCguuggcauag..........................................................................  1     1  seq
....................aggcaagauguuggcauag..........................................................................  1494  0  seq
....................aggcaagauguuggcauaU..........................................................................  20    1  seq
....................aggcaGgauguuggcauag..........................................................................  1     1  seq
....................aCgcaagauguuggcauag..........................................................................  1     1  seq
....................aUgcaagauguuggcauag..........................................................................  1     1  seq
....................aNgcaagauguuggcauagc.........................................................................  1     1  seq
....................aggcaagauguuUgcauagc.........................................................................  5     1  seq
....................aggcaagauguuggAauagc.........................................................................  9     1  seq
....................aggcaagauguuggcaGagc.........................................................................  1     1  seq
....................aggcaagauguuCgcauagc.........................................................................  4     1  seq
....................aggcaagauguuggcGuagc.........................................................................  10    1  seq
```

```
                    Mature                                      Star
gcgaucucagggcacagaccaggcaagauguuggcauagcugauuuacacucugugguccagcucugucgcauauugcccccucugggcccagacuaacaucuuaugugaaaca
....................aUgcaagauguuggcauagc.........................................................................  4      1  seq
....................aggcaagauguuggcauUgc.........................................................................  3      1  seq
....................aggcUagauguuggcauagc.........................................................................  2      1  seq
....................aggcaagauguuggcUuagc.........................................................................  7      1  seq
....................aggcaagauguuggcaCagc.........................................................................  5      1  seq
....................aggcaagauguugUcauagc.........................................................................  2      1  seq
....................aggcaagauguAggcauagc.........................................................................  18     1  seq
....................aggcaagauAuggcauagc..........................................................................  1      1  seq
....................aggcaagauguuggcauagc.........................................................................  40817  0  seq
....................aggcaaAauguuggcauagc.........................................................................  2      1  seq
....................Gggcaagauguuggcauagc.........................................................................  144    1  seq
....................aggcaagGuguuggcauagc.........................................................................  2      1  seq
....................Cggcaagauguuggcauagc.........................................................................  2      1  seq
....................aggcaagaugAuggcauagc.........................................................................  18     1  seq
....................aggcaagauguuggcaAagc.........................................................................  17     1  seq
....................aggcGagauguuggcauagc.........................................................................  6      1  seq
....................agCcaagauguuggcauagc.........................................................................  6      1  seq
....................aggcaagauguuggcauaUc.........................................................................  4      1  seq
....................aggcaagaCguuggcauagc.........................................................................  4      1  seq
....................aggcaagauUuggcauagc..........................................................................  3      1  seq
....................aggcaagauguuggcauGgc.........................................................................  13     1  seq
....................aggcaagauCuggcauagc..........................................................................  3      1  seq
....................aAgcaagauguuggcauagc.........................................................................  16     1  seq
....................aggcaagauguugAcauagc.........................................................................  11     1  seq
....................aCgcaagauguuggcauagc.........................................................................  5      1  seq
....................aggcaagauguuggcauagA.........................................................................  106    1  seq
....................aggcaagauguuggcCuagc.........................................................................  1      1  seq
....................aggcaagaGguuggcauagc.........................................................................  2      1  seq
....................aggcaaUauguuggcauagc.........................................................................  8      1  seq
....................aggcaagauguCggcauagc.........................................................................  5      1  seq
....................aggAaagauguuggcauagc.........................................................................  9      1  seq
....................aggcaagauguuggcauagU.........................................................................  34     1  seq
....................aggcaUgauguuggcauagc.........................................................................  3      1  seq
....................aggUaagauguuggcauagc.........................................................................  1      1  seq
....................aggcaGgauguuggcauagc.........................................................................  4      1  seq
....................aggcaagauguGggcauagc.........................................................................  1      1  seq
....................aggcaagUuguuggcauagc.........................................................................  3      1  seq
....................aggcaagauguuggGauagc.........................................................................  5      1  seq
....................agUcaagauguuggcauagc.........................................................................  7      1  seq
....................Uggcaagauguuggcauagc.........................................................................  3      1  seq
....................aggcaaCauguuggcauagc.........................................................................  1      1  seq
....................aggGaagauguuggcauagc.........................................................................  4      1  seq
....................Nggcaagauguuggcauagc.........................................................................  15     1  seq
....................aggcaagauguuggcauagG.........................................................................  3      1  seq
....................aggcaagauguuAgcauagc.........................................................................  19     1  seq
....................aggcaagaAguuggcauagc.........................................................................  7      1  seq
....................agAcaagauguuggcauagc.........................................................................  13     1  seq
....................aggcaCgauguuggcauagc.........................................................................  2      1  seq
....................aggcaagauguuggUauagc.........................................................................  7      1  seq
....................aggcaagauguugCcauagc.........................................................................  4      1  seq
....................aggcaagaugCuggcauagc.........................................................................  2      1  seq
....................aggcaagauguugCcauagcu........................................................................  4      1  seq
....................aggcaagauguuggcauagcC........................................................................  12     1  seq
....................aggcGagauguuggcauagcu........................................................................  17     1  seq
....................aggGaagauguuggcauagcu........................................................................  7      1  seq
....................aggcaUgauguuggcauagcu........................................................................  6      1  seq
....................aggcaagauguuggcaGagcu........................................................................  6      1  seq
....................Nggcaagauguuggcauagcu........................................................................  25     1  seq
....................aggcaagauAuggcauagcu.........................................................................  12     1  seq
....................Uggcaagauguuggcauagcu........................................................................  12     1  seq
....................aggcaagauguuggcauagcG........................................................................  299    1  seq
....................aggcaagauguuggAauagcu........................................................................  16     1  seq
....................aggcaagauguuggcGuagcu........................................................................  21     1  seq
....................aggcaagauguuggcauGgcu........................................................................  12     1  seq
....................Gggcaagauguuggcauagcu........................................................................  255    1  seq
....................aggcaagauguuUgcauagcu........................................................................  6      1  seq
....................aggcaagauguuggGauagcu........................................................................  16     1  seq
....................aggcaagauguCggcauagcu........................................................................  5      1  seq
....................aggcaagauguuggcUuagcu........................................................................  10     1  seq
....................aggcaagUuguuggcauagcu........................................................................  7      1  seq
```

```
                    Mature                                      Star
gcgaucucagggcacagaccaggcaagauguuggcauagcugauuuacacucuguggucagcucugucgcauauugcccccucugggcccagacuaacaucuuaugugaaaca
....................aggcaCgauguuggcauagcu........................................................................ 1 1 seq
....................aggcaagauguuggcauaUcu........................................................................ 3 1 seq
....................aggcaagaAguuggcauagcu........................................................................ 16 1 seq
....................aggAaagauguuggcauagcu........................................................................ 2 1 seq
....................aggcaagauUuuggcauagcu........................................................................ 3 1 seq
....................aggcaagaugCuggcauagcu........................................................................ 149 1 seq
....................aggcaGgauguuggcauagcu........................................................................ 15 1 seq
....................aggcaagauguuggcaCagcu........................................................................ 5 1 seq
....................aggcaagauguuggcauaAcu........................................................................ 1 1 seq
....................aggcaNgauguuggcauagcu........................................................................ 1 1 seq
....................aUgcaagauguuggcauagcu........................................................................ 16 1 seq
....................agCcaagauguuggcauagcu........................................................................ 21 1 seq
....................aggcaagauguuggcauaCcu........................................................................ 3 1 seq
....................agUcaagauguuggcauagcu........................................................................ 12 1 seq
....................aAgcaagauguuggcauagcu........................................................................ 33 1 seq
....................aggcaagauguuggcauagcu........................................................................ 84476 0 seq
....................agAcaagauguuggcauagcu........................................................................ 17 1 seq
....................aggcUagauguuggcauagcu........................................................................ 8 1 seq
....................aCgcaagauguuggcauagcu........................................................................ 16 1 seq
....................aggcaagauguuggcauagAu........................................................................ 52 1 seq
....................aggcaaAauguuggcauagcu........................................................................ 16 1 seq
....................aggcaagaugGuggcauagcu........................................................................ 2 1 seq
....................aggcaagauguuggUauagcu........................................................................ 26 1 seq
....................aggcaagaCguuggcauagcu........................................................................ 11 1 seq
....................aggcaagGuguuggcauagcu........................................................................ 7 1 seq
....................aggcaagauguAggcauagcu........................................................................ 40 1 seq
....................aggcaagauguuAgcauagcu........................................................................ 56 1 seq
....................aggcaagaGguuggcauagcu........................................................................ 6 1 seq
....................aggcaagauguugAcauagcu........................................................................ 13 1 seq
....................aggcaagauCuuggcauagcu........................................................................ 7 1 seq
....................aggcaaUauguuggcauagcu........................................................................ 7 1 seq
....................aggcaagauguuggcauagcA........................................................................ 131 1 seq
....................aggUaagauguuggcauagcu........................................................................ 16 1 seq
....................aggcaagauguuggcauagGu........................................................................ 1 1 seq
....................aggcaagauguugUcauagcu........................................................................ 8 1 seq
....................aggcaagauguuggcaAagcu........................................................................ 22 1 seq
....................aggcaagauguuCgcauagcu........................................................................ 10 1 seq
....................aNgcaagauguuggcauagcu........................................................................ 1 1 seq
....................aggcaagauguuggcauagUu........................................................................ 10 1 seq
....................aggcaagaugAuggcauagcu........................................................................ 27 1 seq
....................aggcaagauguuUgcauagcug....................................................................... 4 1 seq
....................aggcaagauguuggcauagcuA....................................................................... 273 1 seq
....................aggcaagauguuggcauagcuC....................................................................... 8 1 seq
....................aggcaagauguuggcauagGug....................................................................... 5 1 seq
....................aggcaagUuguuggcauagcug....................................................................... 1 1 seq
....................aggcaagauguuggcauagcAg....................................................................... 16 1 seq
....................aNgcaagauguuggcauagcug....................................................................... 1 1 seq
....................aggcaagauguuggcauaUcug....................................................................... 4 1 seq
....................aggcaaAauguuggcauagcug....................................................................... 5 1 seq
....................aggcaagauguuggUauagcug....................................................................... 5 1 seq
....................aggcaagaugGuggcauagcug....................................................................... 5 1 seq
....................aggcUagauguuggcauagcug....................................................................... 3 1 seq
....................aAgcaagauguuggcauagcug....................................................................... 18 1 seq
....................aggcaagauguCggcauagcug....................................................................... 8 1 seq
....................aggcaagauguuggcauUgcug....................................................................... 3 1 seq
....................aggcaagauguuggcauagcCg....................................................................... 11 1 seq
....................agAcaagauguuggcauagcug....................................................................... 6 1 seq
....................aggcaagauguuggcaCagcug....................................................................... 7 1 seq
....................aggcaagauguuggcauGgcug....................................................................... 8 1 seq
....................aggcaagauguGggcauagcug....................................................................... 3 1 seq
....................aggcaagauguuggGauagcug....................................................................... 6 1 seq
....................Cggcaagauguuggcauagcug....................................................................... 3 1 seq
....................aggcaagaCguuggcauagcug....................................................................... 8 1 seq
....................aggcGagauguuggcauagcug....................................................................... 4 1 seq
....................agCcaagauguuggcauagcug....................................................................... 7 1 seq
....................aggcaagauguugCcauagcug....................................................................... 6 1 seq
....................aggcaagauguuggcauagcGg....................................................................... 99 1 seq
....................aggcaagaugAuggcauagcug....................................................................... 12 1 seq
....................aggcaUgauguuggcauagcug....................................................................... 3 1 seq
....................Gggcaagauguuggcauagcug....................................................................... 166 1 seq
```

```
                    Mature                                      Star
gcgaucucagggcacagaccaggcaagauguuggcauagcugauuuacacucugugggucagcucugucgcauauugcccccucugggcccagacuaacaucuuaugugaaaca
....................aggcaaUauguuggcauagcug.......................................................................	6	1	seq
....................aggcaagauguuggcauagUug.......................................................................	3	1	seq
....................aggcaagauguugUcauagcug.......................................................................	1	1	seq
....................aggGaagauguuggcauagcug.......................................................................	4	1	seq
....................aggcaagauguAggcauagcug.......................................................................	31	1	seq
....................aggcaagauguuggcauagcuU.......................................................................	2681	1	seq
....................aggcCagauguuggcauagcug.......................................................................	1	1	seq
....................Nggcaagauguuggcauagcug.......................................................................	19	1	seq
....................aggcaagauUuuggcauagcug.......................................................................	4	1	seq
....................Uggcaagauguuggcauagcug.......................................................................	7	1	seq
....................agUcaagauguuggcauagcug.......................................................................	9	1	seq
....................aggcaagauguuggcauaAcug.......................................................................	2	1	seq
....................aggcaagauguugAcauagcug.......................................................................	8	1	seq
....................aggcaagauguuggAauagcug.......................................................................	12	1	seq
....................aggUaagauguuggcauagcug.......................................................................	4	1	seq
....................aggcaagauguuggcaGagcug.......................................................................	4	1	seq
....................aggcaagauguuggcauagcug.......................................................................	44039	0	seq
....................aggcaagaAguuggcauagcug.......................................................................	9	1	seq
....................aCgcaagauguuggcauagcug.......................................................................	7	1	seq
....................aggcaagauCuuggcauagcug.......................................................................	3	1	seq
....................aggcaagauguuCgcauagcug.......................................................................	1	1	seq
....................aggcaagauguuggcUuagcug.......................................................................	9	1	seq
....................aggAaagauguuggcauagcug.......................................................................	9	1	seq
....................aUgcaagauguuggcauagcug.......................................................................	7	1	seq
....................aggcaagauAuuggcauagcug.......................................................................	4	1	seq
....................aggcaGgauguuggcauagcug.......................................................................	3	1	seq
....................aggcaagauguuggcaAagcug.......................................................................	15	1	seq
....................aggcaaCauguuggcauagcug.......................................................................	1	1	seq
....................aggcaagauguuggcGuagcug.......................................................................	11	1	seq
....................aggcaagaugCuggcauagcug.......................................................................	6	1	seq
....................aggcaagauguuggcauagAug.......................................................................	30	1	seq
....................aggcaagauguuggcauaCcug.......................................................................	2	1	seq
....................aggcaagauguuAgcauagcug.......................................................................	43	1	seq
....................aggcaagGuguuggcauagcug.......................................................................	8	1	seq
....................agUcaagauguuggcauagcuga......................................................................	101	1	seq
....................aggcaagauguuggcaAagcuga......................................................................	259	1	seq
....................aggcaGgauguuggcauagcuga......................................................................	113	1	seq
....................aggcaagauguuggcauagGuga......................................................................	37	1	seq
....................aggcaagauguuggcCuagcuga......................................................................	7	1	seq
....................aggcaNgauguuggcauagcuga......................................................................	5	1	seq
....................aggcGagauguuggcauagcuga......................................................................	213	1	seq
....................aggcaagauguuggcauagAuga......................................................................	613	1	seq
....................aggcaagauguuggcauCgcuga......................................................................	7	1	seq
....................aggcaaUauguuggcauagcuga......................................................................	76	1	seq
....................aggcaagCuguuggcauagcuga......................................................................	3	1	seq
....................aggcaagGuguuggcauagcuga......................................................................	99	1	seq
....................aggcaagauAuuggcauagcuga......................................................................	73	1	seq
....................aggcaagauguuggcauagcuga......................................................................	888390	0	seq
....................agAcaagauguuggcauagcuga......................................................................	183	1	seq
....................aggcaagauguuggcauaCcuga......................................................................	32	1	seq
....................agCcaagauguuggcauagcuga......................................................................	224	1	seq
....................aggcaagaugGuggcauagcuga......................................................................	35	1	seq
....................aggcCagauguuggcauagcuga......................................................................	4	1	seq
....................aggcaagauguuggcauagUuga......................................................................	71	1	seq
....................aNgcaagauguuggcauagcuga......................................................................	32	1	seq
....................aAgcaagauguuggcauagcuga......................................................................	298	1	seq
....................aggcaagauguugCcauagcuga......................................................................	53	1	seq
....................aggcaagauguuggcGuagcuga......................................................................	189	1	seq
....................aggcaagauguuggcauGgcuga......................................................................	129	1	seq
....................aggcaagauUuuggcauagcuga......................................................................	57	1	seq
....................aggcaagaAguuggcauagcuga......................................................................	157	1	seq
....................aggcaagauCuuggcauagcuga......................................................................	101	1	seq
....................aggcaagauguuggUauagcuga......................................................................	177	1	seq
....................Nggcaagauguuggcauagcuga......................................................................	328	1	seq
....................aggcaagauguuggcauagcCga......................................................................	153	1	seq
....................aCgcaagauguuggcauagcuga......................................................................	125	1	seq
....................aUgcaagauguuggcauagcuga......................................................................	102	1	seq
....................aggcaagauguuUgcauagcuga......................................................................	75	1	seq
....................aggcaagauguuggcauaAcuga......................................................................	62	1	seq
....................aggcaagauguuCgcauagcuga......................................................................	103	1	seq
```

```
                   Mature                     Star
gcgaucucagggcacagaccaggcaagauguuggcauagcugauuuacacucuguggucagcucugucgcauauugcccccucugggcccagacuaacaucuuaugugaaaca
....................agNcaagauguuggcauagcuga......................................................................  2     1  seq
....................aggcaagUuguuggcauagcuga......................................................................  51    1  seq
....................aggcaagauguugUcauagcuga......................................................................  118   1  seq
....................aggcaagauguugAcauagcuga......................................................................  237   1  seq
....................aggcaCgauguuggcauagcuga......................................................................  6     1  seq
....................Uggcaagauguuggcauagcuga......................................................................  114   1  seq
....................aggcaagauguuggcaGagcuga......................................................................  62    1  seq
....................aggcaagauguuggcauagcuUa......................................................................  76    1  seq
....................aggGaagauguuggcauagcuga......................................................................  64    1  seq
....................aggcaagaNguuggcauagcuga......................................................................  1     1  seq
....................aggcaagauguuAgcauagcuga......................................................................  738   1  seq
....................aggcaUgauguuggcauagcuga......................................................................  64    1  seq
....................aggcaagauguuggcauaUcuga......................................................................  73    1  seq
....................aggcaagauguuggcauagcugC......................................................................  33    1  seq
....................aggcaaAauguuggcauagcuga......................................................................  106   1  seq
....................aggcaagaugCuggcauagcuga......................................................................  91    1  seq
....................aggcaagauguuggcauagcuAa......................................................................  500   1  seq
....................aggcaagauguGggcauagcuga......................................................................  17    1  seq
....................aggcaagauguCggcauagcuga......................................................................  154   1  seq
....................aggAaagauguuggcauagcuga......................................................................  143   1  seq
....................aggcaagauguuggcauagcAga......................................................................  355   1  seq
....................aggcaagaGguuggcauagcuga......................................................................  47    1  seq
....................aggUaagauguuggcauagcuga......................................................................  118   1  seq
....................aggcaaCauguuggcauagcuga......................................................................  29    1  seq
....................aggcaagauguuggGauagcuga......................................................................  122   1  seq
....................aggcaagaugAuggcauagcuga......................................................................  374   1  seq
....................aggcaagauguuggAauagcuga......................................................................  205   1  seq
....................aggcaagauguuggcauagcGga......................................................................  103   1  seq
....................aggcaagauguuggcaCagcuga......................................................................  95    1  seq
....................aggcaagauguuggcauUgcuga......................................................................  21    1  seq
....................aggcaagauguuggcauagcuCa......................................................................  110   1  seq
....................aggcaagauguuggcauagcugG......................................................................  6009  1  seq
....................aggcaagauguuggcUuagcuga......................................................................  125   1  seq
....................aggcaagauguuggcauagcugU......................................................................  2754  1  seq
....................aggcUagauguuggcauagcuga......................................................................  44    1  seq
....................Gggcaagauguuggcauagcuga......................................................................  2930  1  seq
....................aggcaagaCguuggcauagcuga......................................................................  99    1  seq
....................aggcaagauguAggcauagcuga......................................................................  492   1  seq
....................Cggcaagauguuggcauagcuga......................................................................  37    1  seq
....................aggcaagauguuggcauagcugaC.....................................................................  10    1  seq
....................aggcaagauguuAgcauagcugau.....................................................................  2     1  seq
....................aggcaagauguuggcauagcuAau.....................................................................  1     1  seq
....................aggcaagauguuggcauagcCgau.....................................................................  2     1  seq
....................aggcaGgauguuggcauagcugau.....................................................................  1     1  seq
....................aggcaagaAguuggcauagcugau.....................................................................  1     1  seq
....................aggcaagauguuggcauagUugau.....................................................................  2     1  seq
....................aggcaagauguAggcauagcugau.....................................................................  1     1  seq
....................aggcaagauguuggcauagcugaN.....................................................................  1     1  seq
....................aggcaagauguuggcauaUcugau.....................................................................  1     1  seq
....................aAgcaagauguuggcauagcugau.....................................................................  1     1  seq
....................aggcaagaGguuggcauagcugau.....................................................................  1     1  seq
....................aggcaagauguuggcGuagcugau.....................................................................  1     1  seq
....................aUgcaagauguuggcauagcugau.....................................................................  1     1  seq
....................aggcaagauguCggcauagcugau.....................................................................  1     1  seq
....................Gggcaagauguuggcauagcugau.....................................................................  9     1  seq
....................aggcaagauguugAcauagcugau.....................................................................  2     1  seq
....................aggcaagauguuggcauGgcugau.....................................................................  1     1  seq
....................aggcaagauguuggAauagcugau.....................................................................  1     1  seq
....................aggGaagauguuggcauagcugau.....................................................................  1     1  seq
....................aggcaagauguuggcauagcugaA.....................................................................  1983  1  seq
....................aggcaagaCguuggcauagcugau.....................................................................  1     1  seq
....................aCgcaagauguuggcauagcugau.....................................................................  2     1  seq
....................aggAaagauguuggcauagcugau.....................................................................  1     1  seq
....................aggcaagauguuggcauagcugUu.....................................................................  664   1  seq
....................Nggcaagauguuggcauagcugau.....................................................................  1     1  seq
....................aggcaagauguuggcauagAugau.....................................................................  2     1  seq
....................aggcaagauguuggcauagcugau.....................................................................  3347  0  seq
....................aggcaagauguuggcauagcugaG.....................................................................  23    1  seq
....................aggcaagauguuggcauagcugauG....................................................................  2     1  seq
....................aggcaagauguuggcauagcugaGu....................................................................  1     1  seq
```

```
                    Mature                                      Star
gcgaucucagggcacagaccaggcaagauguuggcauagcugauuuacacucugugggucagcucugucgcauauugcccccucugggcccagacuaacaucuuaugugaaaca
....................agAcaagauguuggcauagcugauu....................................................................    1     1    seq
....................aggcaagauguuggcaGagcugauu....................................................................    1     1    seq
....................aggcaagauguuggcauagcugauA....................................................................    87    1    seq
....................aggcaagauguAggcauagcugauu....................................................................    1     1    seq
....................aggcaagauguuggcauagcugUuu....................................................................    29    1    seq
....................aggcaagauguugAcauagcugauu....................................................................    1     1    seq
....................aggcaagauguuggcauagcugaAu....................................................................    322   1    seq
....................Gggcaagauguuggcauagcugauu....................................................................    1     1    seq
....................aggcaagauguuggcauagcugauC....................................................................    2     1    seq
....................aggcaagauguuggcauagcuAauu....................................................................    1     1    seq
....................aggcaagauguuAgcauagcugauu....................................................................    1     1    seq
....................aggcaagauguuUgcauagcugauu....................................................................    1     1    seq
....................aggcaagauguuggcauagcugauu....................................................................    806   0    seq
....................aggcaagauguuggcauagcugaCu....................................................................    8     1    seq
....................aggcaagauguuggcauagcugauAu...................................................................    8     1    seq
....................aggcaagauguuggcauagcugauuu...................................................................    3     0    seq
....................aggcaagauguuggcauagcugaAuu...................................................................    16    1    seq
....................aggcaagauguuggcauagcugauCu...................................................................    1     1    seq
....................aggcaagauguuggcauagcugauuuU..................................................................    4     1    seq
....................aggcaagauguuggcauagcugauuuaca................................................................    1     0    seq
....................aggcaagauguuggcauagcugauuuacacu..............................................................    1     0    seq
.....................ggcaagauguuggcauag..........................................................................    1     0    seq
.....................ggcaCgauguuggcauagc.........................................................................    1     1    seq
.....................ggcaagauguAggcauagc.........................................................................    1     1    seq
.....................Agcaagauguuggcauagc.........................................................................    1     1    seq
.....................ggcaagauguuggcauagc.........................................................................    146   0    seq
.....................Ugcaagauguuggcauagcu........................................................................    1     1    seq
.....................ggAaagauguuggcauagcu........................................................................    1     1    seq
.....................ggcaagauguuggcauagcG........................................................................    1     1    seq
.....................ggcaagauguAggcauagcu........................................................................    1     1    seq
.....................Agcaagauguuggcauagcu........................................................................    3     1    seq
.....................ggcaagauguuggcauagcu........................................................................    450   0    seq
.....................ggcaagauguuggcauagcug.......................................................................    78    0    seq
.....................Agcaagauguuggcauagcug.......................................................................    1     1    seq
.....................ggcaagauguuggcauagcuU.......................................................................    4     1    seq
.....................ggcaagauguuggcauagcuga......................................................................    1911  0    seq
.....................Cgcaagauguuggcauagcuga......................................................................    1     1    seq
.....................Agcaagauguuggcauagcuga......................................................................    20    1    seq
.....................ggcaagauguAggcauagcuga......................................................................    2     1    seq
.....................ggcaagauguugCcauagcuga......................................................................    1     1    seq
.....................gAcaagauguuggcauagcuga......................................................................    1     1    seq
.....................ggcaagaugCuggcauagcuga......................................................................    5     1    seq
.....................ggcaagauguuggcauagcugG......................................................................    15    1    seq
.....................ggAaagauguuggcauagcuga......................................................................    1     1    seq
.....................ggcaagauguuggcauagcuAa......................................................................    2     1    seq
.....................ggcaaAauguuggcauagcuga......................................................................    1     1    seq
.....................Ugcaagauguuggcauagcuga......................................................................    1     1    seq
.....................ggcaagauguuAgcauagcuga......................................................................    2     1    seq
.....................ggcaagauguuUgcauagcuga......................................................................    2     1    seq
.....................ggcGagauguuggcauagcuga......................................................................    2     1    seq
.....................ggcaagauguuggUauagcuga......................................................................    2     1    seq
.....................ggcaagaugAuggcauagcuga......................................................................    3     1    seq
.....................ggcaagaCguuggcauagcuga......................................................................    2     1    seq
.....................ggcaagauguuggcauagcugU......................................................................    48    1    seq
.....................ggcaagauguuggcauaAcuga......................................................................    1     1    seq
.....................ggcaagaAguuggcauagcuga......................................................................    2     1    seq
.....................ggcaagauguuggcauagcugaA.....................................................................    19    1    seq
.....................ggcaagauguuggcauagcugaC.....................................................................    1     1    seq
.....................ggcaagauguuggcauagcugau.....................................................................    8     0    seq
.....................Agcaagauguuggcauagcugau.....................................................................    1     1    seq
.....................ggcaagauguuggcauagcugUu.....................................................................    13    1    seq
.....................ggcaagauguuggcauagcugaAu....................................................................    2     1    seq
.....................ggcaagauguuggcauagcugauu....................................................................    11    0    seq
.....................ggcGagauguuggcauagcugauu....................................................................    1     1    seq
.....................ggcaagauguuggcauagcugUuuu...................................................................    1     1    seq
......................gcaagauguuggcauagc.........................................................................    1     0    seq
......................gcaagauguuggcauagcugG......................................................................    1     1    seq
......................gcaagauguuggcauagcuga......................................................................    3     0    seq
.......................caagauguuggcauagcu........................................................................    1     0    seq
.......................caagauguuggcauagcuga......................................................................    9     0    seq
```

Mature Star

```
gcgaucucagggcacagaccaggcaagauguuggcauagcugauuuacacucuguggucagcucugucgcauauugcccccucugggcccagacuaacaucuuaugugaaaca
........................aagauguuggcauagcuga......................................................................	9	0	seq
.........................agauguuggcauagcuga......................................................................	14	0	seq
.........................................gauuuacacucuguggucag....................................................	1	0	seq
..................................................ucuguggucagcucugucgcauauugcccc.................................	1	0	seq
..................................................ucuguggucagcucugucgcauauugccccc................................	1	0	seq
.........................................................ucagcucugucgcauauugcc...................................	2	0	seq
.........................................................ucagcucugucgcauauugccccc................................	2	0	seq
..........................................................cagcucugucgcauauugccc..................................	8	0	seq
..........................................................cagcucugucgcauauugcccU.................................	8	1	seq
..........................................................cagcucugucgcauauugcccc.................................	5	0	seq
..........................................................cagcucugucgcauauugccccU................................	12	1	seq
..........................................................cagcucugucgcauauugcccccu...............................	1	0	seq
...........................................................agcucugucgcauauugc....................................	5	0	seq
...........................................................Ggcucugucgcauauugcc...................................	1	1	seq
...........................................................agcucugucgcauauugcc...................................	114	0	seq
...........................................................agcucugucgcauauugccU..................................	10	1	seq
...........................................................agcucugucgcauauugccA..................................	1	1	seq
...........................................................agcucugucgcauauugccc..................................	163	0	seq
...........................................................Ggcucugucgcauauugccc..................................	1	1	seq
...........................................................agcucugucgcauauugccG..................................	1	1	seq
...........................................................agcucugucgcauauugcccU.................................	97	1	seq
...........................................................agcucugucgcauauugUccc.................................	1	1	seq
...........................................................agcucugucgcauauugcccc.................................	362	0	seq
...........................................................agcucuguUgcauauugcccc.................................	1	1	seq
...........................................................agcucCgucgcauauugcccc.................................	1	1	seq
...........................................................agcucugucgcauauAgcccc.................................	1	1	seq
...........................................................agcucugucgcauauugcccUc................................	1	1	seq
...........................................................agcucugucgcaCauugccccc................................	1	1	seq
...........................................................agcucuCucgcauauugccccc................................	1	1	seq
...........................................................agcucugucgcauauugcUccc................................	2	1	seq
...........................................................agcucugucgcauauugccUcc................................	1	1	seq
...........................................................agcAcugucgcauauugccccc................................	1	1	seq
...........................................................agcucugucgcauauugcccAc................................	1	1	seq
...........................................................agcucuguUgcauauugccccc................................	1	1	seq
...........................................................agcucugucgcauauugccAcc................................	8	1	seq
...........................................................agcucugucgcauauCgccccc................................	1	1	seq
...........................................................agcucugucgcaAauugccccc................................	1	1	seq
...........................................................agcucugucgcauauugUcccc................................	1	1	seq
...........................................................agcucugucgcUuauugccccc................................	1	1	seq
...........................................................agcucAgucgcauauugccccc................................	1	1	seq
...........................................................agcucugucgcauauugccccA................................	5	1	seq
...........................................................agcucugucgcauUuugccccc................................	1	1	seq
...........................................................agcucugucgcauauugccccc................................	1304	0	seq
...........................................................agcucugucgcauauugccccU................................	87	1	seq
...........................................................agcucugucgcauauAgccccc................................	1	1	seq
...........................................................agcucugCcgcauauugccccc................................	1	1	seq
...........................................................Ggcucugucgcauauugccccc................................	11	1	seq
...........................................................agcucugucgcauauugcccAcu...............................	1	1	seq
...........................................................agcucugucgcauauugcccccA...............................	6	1	seq
...........................................................agcucugucgcauauugcccccu...............................	157	0	seq
...........................................................agcucugucgcauauugccUccu...............................	1	1	seq
...........................................................Ggcucugucgcauauugcccccu...............................	2	1	seq
...........................................................agcucugucgcauauugccccUu...............................	6	1	seq
...........................................................agcucugucgcauauugcccccC...............................	1	1	seq
...........................................................agcucugucgcauauugcccccuU..............................	11	1	seq
............................................................gcucugucgcauauugcc...................................	5	0	seq
............................................................gcucugucgcauauugcccc.................................	1	0	seq
............................................................gcucugucgcauauugccccU................................	1	1	seq
............................................................gcucugucgcauauugccccc................................	53	0	seq
............................................................gcucugucgcauauugcccccu...............................	15	0	seq
.............................................................cucugucgcauauugcccc.................................	4	0	seq
.............................................................cucugucgcauauugccccc................................	28	0	seq
.............................................................cucugucgcauauugccccU................................	5	1	seq
.............................................................cuAugucgcauauugcccccu...............................	1	1	seq
.............................................................cucugucgcauauugcccccu...............................	290	0	seq
.............................................................cucugucgcauauugcccccG...............................	3	1	seq
.............................................................cucugucgcauauugccccUu...............................	3	1	seq
.............................................................cucugucgcauauugcccUcu...............................	1	1	seq
.............................................................cucugucgcauauugcccccA...............................	4	1	seq
.............................................................cucugucgcauauugcccccuA..............................	2	1	seq
```

```
                    Mature                                      Star
gcgaucucagggcacagaccaggcaagauguuggcauagcugauuuacacucugugguc agcucugucgcauauugcccccucugggcccagacuaacaucuuaugugaaaca
.............................................................cucugucgcauauugcccccuU..............................   6     1     seq
.............................................................cucugucgcauauugcccccuc..............................   1     0     seq
..............................................................ucugucgcauauugccccc................................   1     0     seq
..............................................................Ccugucgcauauugcccccu...............................   1     1     seq
..............................................................ucugucgcauauugcccccA...............................   7     1     seq
..............................................................ucugucgcauauugcccccu...............................   128   0     seq
..............................................................ucugucgcauauugcccccuU..............................   2     1     seq
..............................................................ucugucgcauauugcccccuc..............................   2     0     seq
..............................................................ucugucgcauauugcccccuUu.............................   2     1     seq
```
